# Inflammation index SIRI is associated with increased all-cause and cardiovascular mortality among patients with hypertension

**DOI:** 10.3389/fcvm.2022.1066219

**Published:** 2023-01-11

**Authors:** Songfeng Zhao, Siyuan Dong, Yongkai Qin, Yutong Wang, Baorui Zhang, Aihua Liu

**Affiliations:** ^1^Department of Neurosurgery, The Third Xiangya Hospital, Central South University, Changsha, China; ^2^Department of Neurosurgery, Beijing Tongren Hospital, Capital Medical University, Beijing, China; ^3^Beijing Neurosurgical Institute, Beijing Tiantan Hospital, Capital Medical University, Beijing, China

**Keywords:** National Health and Nutrition Examination Survey (NHANES), hypertension, inflammation, systemic inflammatory response index (SIRI), mortality

## Abstract

**Background:**

Inflammation plays an essential role in the pathogenesis of hypertension. A novel inflammatory biomarker systemic inflammatory response index (SIRI) is related with all-cause and cardiovascular (CVD) mortality, while the role of SIRI in hypertension patients is unclear.

**Methods:**

A total of 21,506 participants with hypertension were recruited in the National Health and Nutrition Examination Survey (NHANES) from 1999 to 2018. SIRI was calculated as the neutrophil count ^*^ monocyte count/lymphocyte count. Hypertension was defined according to the examination of blood pressure, prescription, and self-reported physician diagnosis. Survival status was followed through 31 December 2019. The non-linear relationship was assessed using restricted cubic spline analysis. The association of all-cause mortality with SIRI was evaluated using the Kaplan–Meier curve and the weighted Cox regression analysis. The predictive abilities were assessed with Receiver operating curve.

**Results:**

During 189,063 person-years of follow-up, 5,680 (26.41%) death events were documented, including 1,967 (9.15%) CVD related deaths. A J-shaped association was observed between SIRI and all-cause and CVD mortality. The Kaplan–Meier curve indicated the all-cause and CVD mortality risks were higher in high SIRI quartiles compared with lower SIRI quartiles. After adjusting for all covariates, the SIRI was positively associated with the all-mortality risk with HR = 1.19 (1.15, 1.22), and CVD mortality with HR = 1.19 (1.15, 1.24). The result was robust in subgroup analysis and sensitivity analysis.

**Conclusion:**

Elevated SIRI level is associated with increased all-cause and CVD mortality among patients with hypertension. SIRI is considered as a potential inflammatory biomarker in the clinical practice. Further large-scale cohort studies are required to confirm our findings.

## Introduction

Hypertension has become a severe public health problem, with an estimation of over 30% adults aged between 30 and 79 living with hypertension worldwide in 2019 (1). Individuals with hypertension have a higher risk of developing cardiovascular disease (CVD) and renal disease compared with those without hypertension (2). Moreover, increased all-cause mortality was reported in hypertension people (3).

Extensive studies have shown that inflammation plays an essential role in the pathogenesis of hypertension (4–7). SIRI has been considered a good index that could reflect the human body's chronic inflammatory status. It can predict the prognosis of patients with stroke and cancer (8–10). However, few studies have directly investigated the role of SIRI among hypertension patients.

Therefore, in this study, we aimed to examine the association between inflammation reflected by SIRI and all-cause and CVD mortality among a nationally representative sample of the U.S. population with hypertension. We assumed that an elevated SIRI would be associated with a higher risk of all-cause and CVD mortality in hypertensive patients.

## Subjects and methods

### Study population

Data were obtained from the National Health and Nutrition Examination Survey (NHANES), which is a national survey that collected nutritional and health status across non-institutional civilians of the U.S. This project is conducted by the National Center for Health Statistics (NCHS) at the Centers for Disease Control and Prevention (CDC). All NHANES protocols were approved by the NCHS ethics review board (ERB) and written informed consents were acquired from the participants' legal guardians or next of kin. The study conforms to the STROBE guideline.

**Figure 1:**
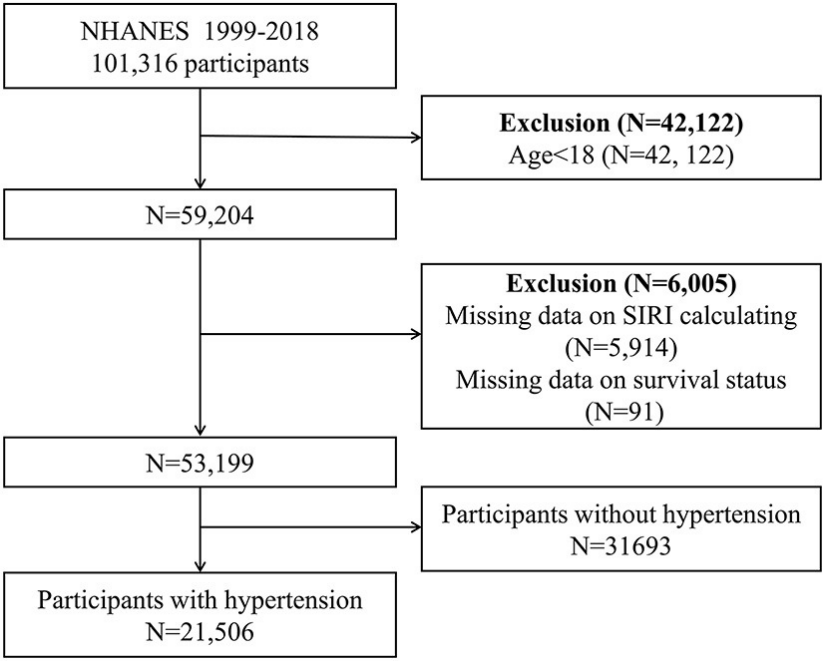
Flow chart of the sample collection.

We combined data from 10 NHANES cycles (1999–2018). The survival status was ascertained by linkage to the National Death Index (NDI) through 31 December 2019. CVD mortality was defined as ICD-10 codes I00–I09, I11, I13, I20–I51, or I60–I69 according to ICD-10 code. At first, 101,316 participants were included, after exclusion people with age < 18 (*n* = 42,122), missing complete data for SIRI calculation (*n* = 5,914), missing survival status (*n* = 91), and without hypertension (*n* = 31,693), a total of 21,506 participants were enrolled in our study (Figure 1).

### Diagnosis of hypertension

We define hypertension as follows: (1) the average systolic blood pressure (SBP) is over 140 mmHg or the average diastolic blood pressure (DBP) is over 90 mmHg, (2) self-reported diagnosis by physician. (3) Prescription of anti-hypertensive drugs.

### Calculation of SIRI

The calculation of SIRI is shown below, while N represents neutrophil counts, M represents monocyte counts, and L represents lymphocyte counts. The units of N, M, and L were all 1,000 cells/μl. Data were collected from the complete blood count with five-part differential whole blood.


$$\begin{array}{l} SIRI=\text{N }*\text{ M}/\text{L} \end{array}$$

### Assessment of covariates

Continuous covariates include age, family-to-poverty ratio, and SIRI. Categorial variates include sex (male/female), race (white, black, others), education (<high school/≥high school), diabetes mellitus (yes/no), cardiovascular disease (yes/no), hyperlipidemia (yes/no), chronic kidney disease (yes/no), smoking status (current, former, never), drinking status (current, former, never), BMI (<30 kg/m^2^/≥30 kg/m^2^), and prescription medication use of anti-hypertensive, hypoglycemic, and lipid-lowering medications.

Smoking status was categorized into never smoker (smoked less than 100 cigarettes in life), former smoker (smoked over 100 cigarettes but not still smoking recently), and current smoker. Drinking status was divided into never drinker (drunk <12 times in a lifetime), former drinker (drunk ≥12 times in life but did not drink last year), and current drinker (drunk ≥12 times in a lifetime and still drinking recently). We defined diabetes mellitus (DM) if any of these criteria is met: (1) doctor told you have diabetes, (2) glycohemoglobin HbA1c (%) >6.5, (3) fasting glucose (mmol/L) ≥7.0, (4) random blood glucose (mmol/L) ≥11.1, (5) 2-h OGTT blood glucose (mmol/L) ≥11.1, (6) use of diabetes medication or insulin. We defined hyperlipidemia (HLD) as a self-reported diagnosis by doctors or a prescription of anti-dyslipidemia drugs. Cardiovascular disease (CVD) was recognized by self-reported physician's diagnosis of congestive heart failure, coronary heart disease, angina, heart attack, or stroke. The estimated glomerular filtration rate (eGFR) was estimated using the CKD-EPI formula (11). Chronic kidney disease (CKD) is defined as eGFR < 60 ml/min/1.73 m^2^ or albuminuria ≥30 mg/g (12).

### Statistical analysis

We carried out all statistical analyses under the instruction of CDC analytical guidelines. We used the R package “survey” to carry out complex survey analyses with proper weights. The continuous variates are presented as the mean (standard error, SE), while categorial variates are presented as the proportion (95% confidence interval, 95%CI). The differences of people with different SIRI quartiles were employed by weighted *t*-test for continuous variates and weighted χ^2^ test for categorical variates.

To explore the potential linear relationship between SIRI and all-cause and CVD mortality. We carried out weighted five-knots (5th, 27.5th, 50th, 72.5th, and 95th percentiles) restricted cubic spline (RCS) with adjusted covariates. Nonlinearity was tested using the likelihood ratio test. Kaplan–Meier methodology was used to construct the survival curves. We performed weighted multivariable cox proportional hazards regression analyses to evaluate the association between SIRI and all-cause and CVD mortality. The results were shown as hazard ratios (HR) and 95%CI. Three models were constructed: model 1 was the crude model; model 2 adjusted for age, sex, and race; model 3 was further adjusted for BMI, SBP, DBP, education level, income-to-poverty ratio, smoking status, alcohol intake, personal history of CVD, CKD, DM, and HLD.

Besides, to compare the predictive ability of SIRI with traditional inflammatory biomarkers such as neutrophil-lymphocyte ratio (NLR), monocyte, and platelet-lymphocyte ratio (PLR), we further constructed receiver operating characteristic (ROC) analysis and calculated the area under curve (AUC) for each predictor. DeLong test was used to determine the difference of ROCs.

We carried out the subgroup analysis with potential confounding factors. The effects of different subgroups and the significance of interaction were calculated and shown by forest plot. We conducted several sensitivity analyses to determine the robustness. (1) We divided the SIRI into quartiles to transform the SIRI as a categorical variable. (2) We repeated the main analyses according to tertiles of the SIRI level. (3) We excluded patients who died within 2 years of follow-up. (4) We exclude participants with abnormal total leukocyte count (<4,000 cells/μl or >11,000 cells/μl). (5) To adjust the effect of medication, we then further adjusted for use of anti-hypertensive, hypoglycemic, and lipid-lowering medications in model 4. (6) We further adjusted for C-reactive protein (CRP) in model 5.

R package “missForest” were used to impute the missing data of covariates by using the random forest algorithm (13). All statistical analyses were performed using R software (version 3.6.1). *P*-value < 0.05 was considered statistically significant.

## Results

During 189,063 person-years of follow-up, 5,680 (26.41%) death events were documented, including 1,967 (9.15%) CVD related deaths. The median of follow-up time was 97 months. The weighted baseline characteristics of 21,506 participants according to SIRI quartiles (Q1: 0.03–0.75; Q2: 0.75–1.12; Q3: 1.12–1.66; Q4: 1.66–24.6) are shown in Table 1. Generally, participants with higher SIRI tend to be older with a higher prevalence of CVD, CKD, and DM. Besides, samples with higher SIRI are also more likely to be male and obese. The all-cause mortality increased with elevated SIRI quartiles from 12.22% in Q1 to 26.29% in Q4, while the CVD mortality increased from 4.87% in Q1 to 11.21% in Q4.

**Table 1:** Weighted baseline characteristics of participants with hypertension according to SIRI quantiles.

**Characteristics**	**Quartile of SIRI**				***P*-value**
	**Quartile 1**	**Quartile 2**	**Quartile 3**	**Quartile 4**	
SIRI	0.55 (0.00)	0.94 (0.00)	1.37 (0.00)	2.59 (0.02)	<0.0001
Age	54.21 (0.31)	56.01 (0.28)	57.58 (0.28)	59.90 (0.33)	<0.0001
**Sex**					
Male	40.11 (0.88)	45.36 (1.03)	51.31 (1.10)	58.24 (1.09)	<0.0001
Female	59.89 (0.88)	54.64 (1.03)	48.69 (1.10)	41.76 (1.09)	
**Race/ethnicity**					
White	53.31 (1.73)	70.08 (1.38)	75.50 (1.26)	79.84 (1.15)	<0.0001
Black	27.06 (1.40)	12.17 (0.83)	8.80 (0.66)	6.73 (0.52)	
Others	19.64 (1.06)	17.75 (0.99)	15.71 (0.94)	13.44 (0.91)	
**Education**					
≥high school	78.95 (0.88)	82.03 (0.82)	81.64 (0.79)	80.66 (0.81)	0.012
< high school	21.05 (0.88)	17.97 (0.82)	18.36 (0.79)	19.34 (0.81)	
Poverty	2.86 (0.04)	3.03 (0.04)	2.91 (0.03)	2.83 (0.03)	<0.0001
**BMI (kg/m** ^2^ **)**					
< 30	55.27 (1.05)	52.60 (1.03)	49.50 (1.03)	49.93 (0.92)	<0.001
≥30	44.73 (1.05)	47.40 (1.03)	50.50 (1.03)	50.07 (0.92)	
SBP (mmHg)	133.65 (0.36)	134.36 (0.33)	134.37 (0.36)	134.52 (0.38)	0.17
DBP (mmHg)	75.52 (0.29)	75.08 (0.28)	73.78 (0.30)	72.25 (0.34)	<0.0001
**Smoking status**					
Current	17.40 (0.77)	16.46 (0.74)	19.99 (0.78)	21.87 (0.79)	<0.0001
Former	27.73 (1.01)	30.20 (0.83)	32.45 (0.95)	34.35 (0.88)	
Never	54.87 (1.09)	53.34 (1.01)	47.56 (0.94)	43.79 (1.02)	
**Drinking status**					
Current	65.99 (1.24)	67.69 (1.21)	67.13 (1.04)	65.18 (1.01)	<0.001
Former	18.15 (0.91)	17.86 (0.91)	19.74 (0.77)	22.19 (0.75)	
Never	15.86 (0.81)	14.46 (0.70)	13.13 (0.68)	12.63 (0.67)	
**CVD**					
No	86.65 (0.66)	85.70 (0.68)	81.57 (0.65)	75.18 (0.81)	<0.0001
Yes	13.35 (0.66)	14.30 (0.68)	18.43 (0.65)	24.82 (0.81)	
**CKD**					
No	80.24 (0.74)	76.15 (0.81)	73.85 (0.85)	63.62 (0.91)	<0.0001
Yes	19.76 (0.74)	23.85 (0.81)	26.15 (0.85)	36.38 (0.91)	
**DM**					
No	77.11 (0.82)	78.50 (0.77)	74.41 (0.77)	69.09 (0.91)	<0.0001
Yes	22.89 (0.82)	21.50 (0.77)	25.59 (0.77)	30.91 (0.91)	
**HLD**					
No	20.44 (0.87)	18.91 (0.87)	16.66 (0.77)	17.27 (0.80)	0.006
Yes	79.56 (0.87)	81.09 (0.87)	83.34 (0.77)	82.73 (0.80)	
N (1,000 cells/μl)	2.97 (0.02)	3.90 (0.02)	4.66 (0.02)	6.04 (0.03)	<0.0001
M (1,000 cells/μl)	0.44 (0.01)	0.53 (0.00)	0.61 (0.00)	0.75 (0.00)	<0.0001
L (1,000 cells/μl)	2.14 (0.01)	2.48 (0.05)	2.21 (0.02)	2.09 (0.02)	<0.0001
**All-cause death**					
No	87.78 (0.63)	86.72 (0.70)	82.48 (0.58)	73.71 (0.92)	<0.0001
Yes	12.22 (0.63)	13.28 (0.70)	17.52 (0.58)	26.29 (0.92)	
**CVD related death**					
No	95.13 (0.35)	94.99 (0.35)	93.14 (0.44)	88.79 (0.58)	<0.0001
Yes	4.87 (0.35)	5.01 (0.35)	6.86 (0.44)	11.21 (0.58)	

SIRI, systemic inflammatory response index; BMI, body mass index; SBP, systolic blood pressure; DBP, diastolic blood pressure; CVD, cardiovascular disease; CKD, chronic kidney disease; HLD, hyperlipidemia; N, neutrophil; M, monocyte; L, lymphocyte.

**Figure 2:**
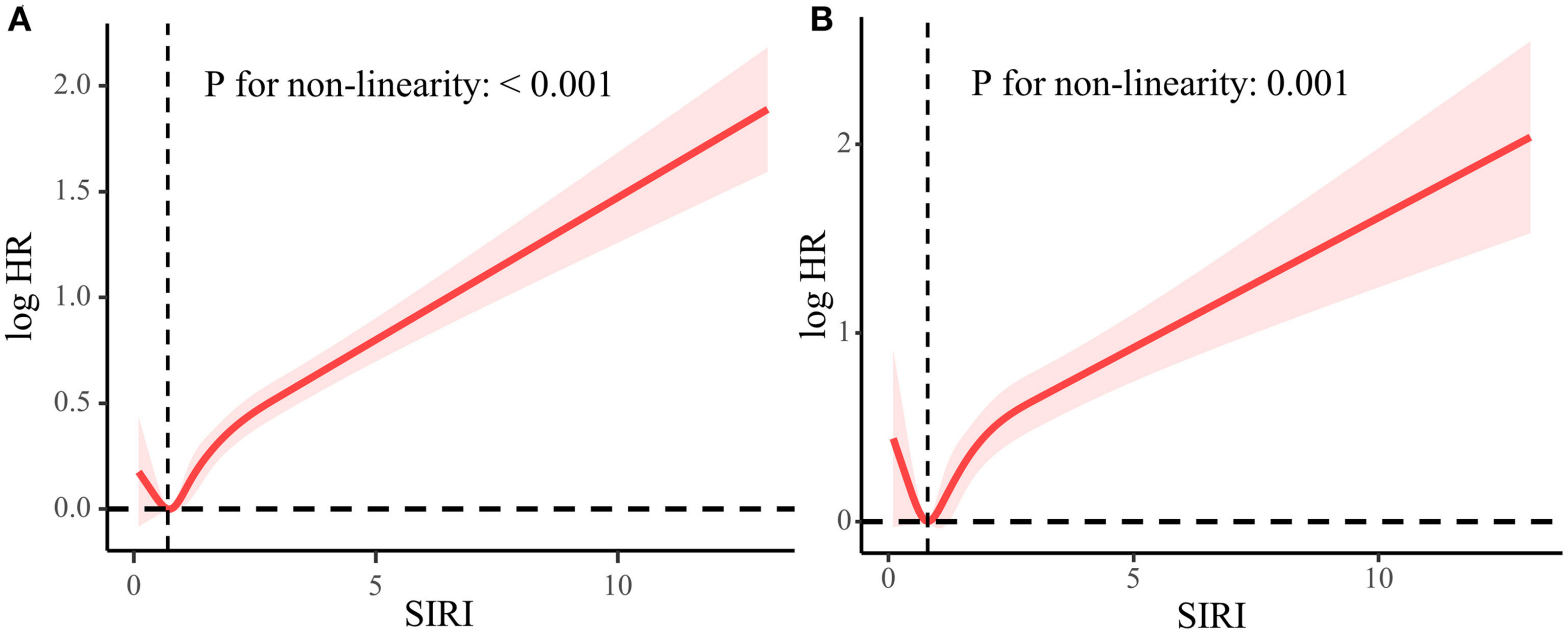
Association between SIRI and all-cause mortality **(A)** and CVD mortality **(B)** used five-knots RCS curve adjusted for all covariates. The dashed vertical lines represent the SIRI level of 0.75 and 0.82, respectively, which is of the lowest HR.

**Figure 3:**
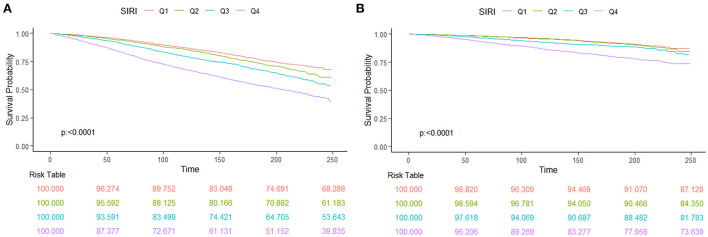
The Kaplan–Meier curve and risk table for survival probabilities from all-cause mortality **(A)** and CVD mortality **(B)** of hypertension patients.

We found a “J” shape relationship between SIRI and all-cause and CVD mortality among hypertension patients, which is shown in Figure 2. The RCS curve revealed that the risk of all-cause mortality decreased until SIRI reached around 0.75 and then increased thereafter (*P* for non-linearity < 0.001). Similar trend was found in the relationship between SIRI and CVD mortality with the change point at 0.82 (*P* for non-linearity = 0.001). The Kaplan–Meier survival curves indicated the survival probability decreased significantly with the increase of SIRI quartiles (Figure 3).

Weighted Cox proportional hazards regression analyses confirmed that SIRI is positively related with all-cause and CVD mortality among hypertension patients (Tables 2, 3). In model 1, the continuous SIRI revealed a 30% (95% CI, 1.25–1.35) higher hazard of death from all-cause and cardiovascular disease with each 1-unit increase of SIRI. The HR for Model 2 and Model 3 were 1.21 (1.16, 1.26) and 1.18 (1.15, 1.21), respectively. Besides, after adjusting for all confounders, the risk of all-cause mortality increased in SIRI Q3 and Q4 groups with the HRs of 1.17 (1.06, 1.28) and 1.55 (1.41, 1.70) compared with the Q1 group. The HR for CVD mortality after fully adjustment was 1.19 (1.15, 1.24) and the highest quartile of SIRI was related to a 60% higher risk CVD mortality.

**Table 2:** Risk of all-cause mortality among hypertension patients according to SIRI.

	**Model 1**	**Model 2**	**Model 3**
	**HR (95% CI)**	**HR (95% CI)**	**HR (95% CI)**
Continuous	**1.30 (1.25, 1.35)**	**1.21 (1.16, 1.26)**	**1.18 (1.15, 1.21)**
**Categories**			
Q1	Reference	Reference	Reference
Q2	1.10 (0.97, 1.24)	1.04 (0.93, 1.16)	1.03 (0.93, 1.13)
Q3	**1.53 (1.36, 1.72)**	**1.31 (1.17, 1.46)**	**1.17 (1.06, 1.28)**
Q4	**2.72 (2.41, 3.09)**	**1.97 (1.76, 2.20)**	**1.55 (1.41, 1.70)**
*P* for trend	<0.0001	<0.0001	<0.0001

Model 1: unadjusted. Model 2: adjusted for age, sex, race. Model 3: further adjusted for education, income to poverty, BMI, SBP, DBP, smoking status, drinking status, CVD, CKD, DM, and HLD.

**Table 3:** Risk of CVD mortality among hypertension patients according to SIRI.

	**Model 1**	**Model 2**	**Model 3**
	**HR (95% CI)**	**HR (95% CI)**	**HR (95% CI)**
Continuous	**1.30 (1.25, 1.35)**	**1.20 (1.14, 1.25)**	**1.19 (1.15, 1.24)**
**Categories**			
Q1	Reference	Reference	Reference
Q2	1.05 (0.90, 1.24)	0.96 (0.83, 1.11)	0.91 (0.80, 1.04)
Q3	**1.52 (1.25, 1.84)**	**1.23 (1.03, 1.46)**	1.08 (0.92, 1.28)
Q4	**2.90 (2.43, 3.46)**	**1.99 (1.67, 2.37)**	**1.60 (1.35, 1.89)**
*P* for trend	<0.0001	<0.0001	<0.0001

Model 1: unadjusted. Model 2: adjusted for age, sex, race. Model 3: further adjusted for education, income to poverty, BMI, SBP, DBP, smoking status, drinking status, CVD, CKD, DM, and HLD.

**Figure 4:**
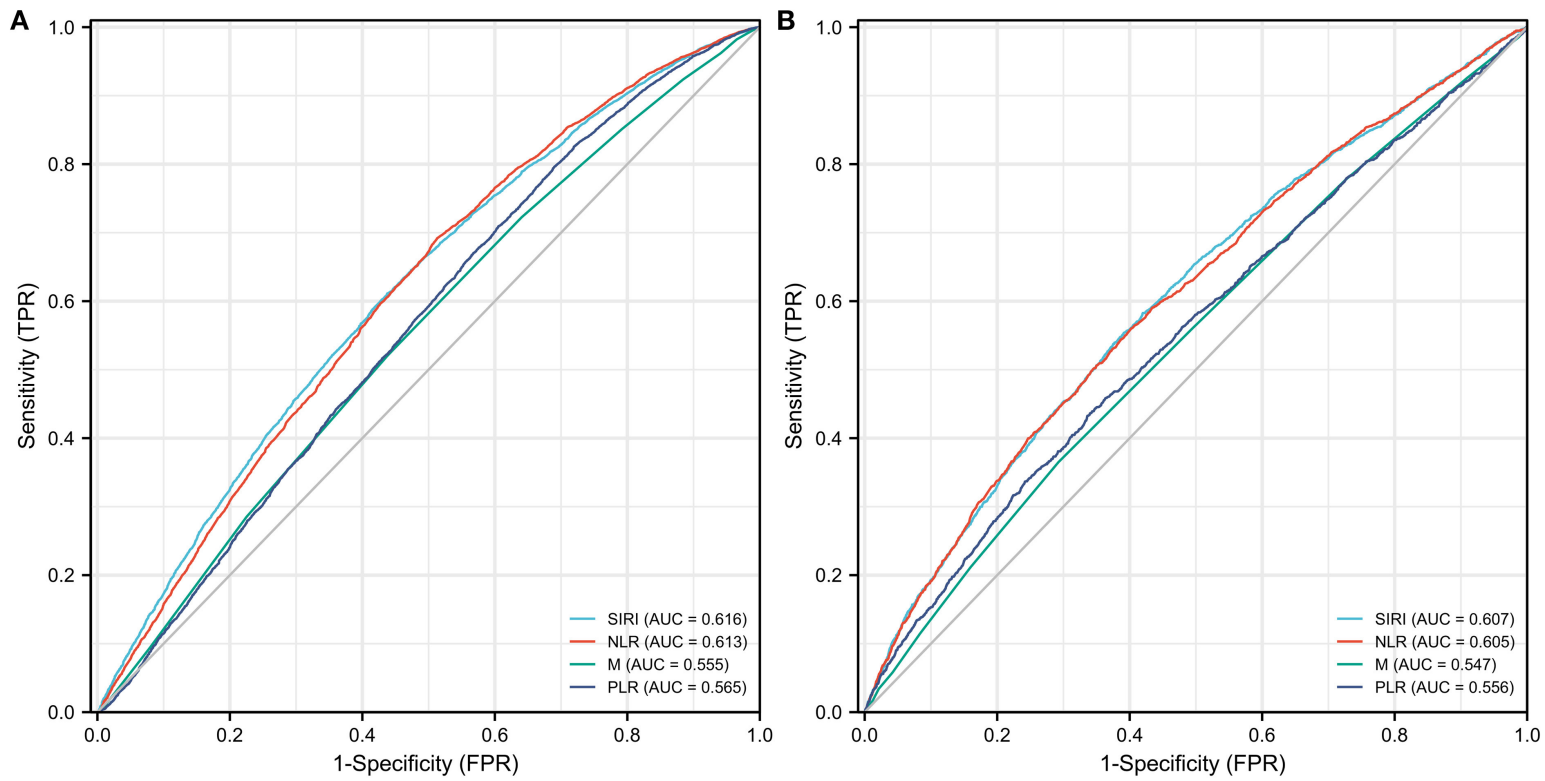
Comparison for the predictive ability of SIRI and traditional inflammatory biomarkers for all-cause mortality **(A)** and CVD mortality **(B)**.

In the ROC analyses (Supplementary Table 1, Figure 4), SIRI showed a higher AUC (0.616) for predicting all-cause mortality compared to NLR (0.613), M (0.555), and PLR (0.565), but the differences were not statistically significant. With respect to CVD mortality, SIRI still displayed the highest AUC (0.607) in comparison to NLR (0.605), M (0.547), and PLR (0.556), although not significantly. Interestingly, the cut-off value of SIRI was determined as 1.231 for both outcomes.

The same trends were also observed in the subgroup analyses stratified by confounders (Supplementary Figure 1). There were stronger associations for participants who were female, former drinkers, with obesity and higher income (*P* for interaction < 0.05). Notably, subjects with CKD seem more likely to be influenced by the SIRI although not statistically significant (*P* = 0.0746). The correlations were generally robust in sensitivity analyses when excluded participants who died within 2 years of follow-up, with abnormal total leukocyte count, further adjusted for medication use and CRP level, or repeating the main analyses by tertiles of SIRI (Supplementary Tables 2–4).

## Discussion

In this retrospective cohort study, we found that the SIRI was independently associated with all-cause and CVD mortality among hypertension subjects. This correlation seems more remarkable among subjects with female sex, higher income, obesity, and history of drinking. Our finding suggested that higher inflammatory status reflected by SIRI was an independent risk factor for all-cause and CVD mortality in hypertension subjects.

Nonlinear relationships between SIRI and all-cause and cardiovascular mortality were observed in the weighted RCS. Consistent to our study, several studies have demonstrated J shaped relationship between white blood cell (WBC) count and all-cause and CVD mortality (14–16). According to our study, the turning points of SIRI were 0.75 and 0.82 for all-cause and CVD mortality, respectively. Notably, a cohort study involving 436,750 Chinese adults reported that the lowest adjusted HRs of all-cause and CVD mortality of monocyte and NLR were found in the second decile of 10 deciles (17), which corresponds to our findings. Conversely, Han et al. (18) indicated SIRI was always positively associated major adverse cardiovascular events. This may be a result of the differences in race and inclusion criteria. Although the specific mechanism for J shaped relationship is still elusive, a hypothesis is that an extremely low level of WBC may indicate a weakened immune system and poor overall health.

Strong evidence has proved the inflammatory process in hypertension is dominated by both innate and adaptive immune responses (19). Activated immune cells can infiltrate target tissue and lead to promoting end-organ damage via excessive secretion of cytokines and chemokines. Notably, monocyte, one component of SIRI, is critical in the development of inflammation and hypertension (20). We also found a positive association between monocyte and all-cause and CVD mortality (Supplementary Table 6), which indicated that monocytes played a critical role in the pathogenesis of hypertension (HR = 1.28 for all-cause mortality, HR = 1.19 for CVD mortality). During the past decades, substantial studies have focused on the predictive values of inflammatory indicators in hypertension (21–25). Maio et al. (25) reported that high sensitivity-CRP (hs-CRP) was independently associated with heart failure in hypertensive patients (HR = 1.162, 95% CI = 1.072–1.259). Previous studies have shown that WBC and NLR significantly increased in hypertension patients, and the NLR kept increasing with the prolongation of the duration of hypertension (26). Similarly, a recent population-based study also indicated that NLR could be a predictor of renal damage in the hypertension population (27). Furthermore, several animal experiments also suggested that inflammatory indicators may influence the outcome of hypertension (28–30). Wenzel et al. (28) found depletion of monocyte can keep lower blood pressure and protect the mice from vascular dysfunction. Likewise, Mice lacking TNFα and IL-6 also exhibited decreased blood pressure (29, 30).

Despite SIRI being considered as a novel inflammatory biomarker, it is more comprehensive, easily accessible, and broadly validated in several studies (8, 10, 18). It can reflect the inflammatory and immunological status of the human body. Besides, SIRI was reported as a better predictor of stroke prognosis compared to classic inflammatory indicators, such as NLR, PLR and monocyte-lymphocyte ratio (MLR) (8). For example, Zhang et al. (8) demonstrated that SIRI had a better predictive performance of stroke prognosis over PLR, NLR, and MLR with receiver operating curve (ROC) analysis. Similarly, SIRI was considered as a more promising inflammatory biomarker than NLR and MLR among acute coronary syndrome patients (18). Our results also revealed that the predictive abilities of SIRI were comparable to traditional inflammatory biomarkers, such as NLR, M, and PLR. Although the differences were not statistically significant in our research, SIRI, as a more comprehensive indicator, may surpass traditional indicators in other populations. Further studies are still needed to investigate the predictive ability and suitable threshold range of SIRI in different participants.

Recently a cohort study in China illustrated the positive correlation between SIRI and risk of all-cause mortality with an adjusted HR = 1.393 (1.296, 1.498) (31). However, the participants are all from an occupational population in China which is not representative and evidence for US civilization is limited. Besides, the author only applied subgroup analysis by gender or age. An observational study enrolled 1,724 participants with acute coronary syndrome undergoing percutaneous coronary intervention indicated that SIRI can predict major adverse cardiovascular events (18). Our results are consistent with previous studies on the relationship between SIRI and poor prognosis.

In subgroup analysis, we found the estimates of SIRI were higher among obese and female participants. It's well accepted that the prevalence of hypertension was lower among females in the early stage and inversed after menopause (32). Some antihypertensive drugs also showed worse effect among females (33). The sexual differences may be owing to the intersection of hormone and immunological system (34). Obesity is strongly associated with the development of hypertension. Obese hypertensives are prone to have poor prognosis because of excessive inflammation, insulin resistance, and activation of the renin-angiotensin-aldosterone axis (35, 36). As a result, weight reduction through healthy behavior or medicine is extremely important for obese hypertensives (36, 37).

To the best of our knowledge, this is the first study to explore the relationship between SIRI and all-cause mortality among patients with hypertension. There are several strengths of our study. First, this study is a large population cohort study enrolled 21,506 participants with hypertension, which represent 78,846,943 samples. Second, we also adjusted for possible confounders affecting the mortality including personal habits, personal history, and socioeconomic status. Third, we conducted a series of sensitivity analyses to confirm the robustness of the relationship. However, there are also some limitations to be considered. First, we cannot infer the causality attributed to the observational study design. Further large-sample prospective studies are still in need. Second, we calculated SIRI with one-time complete blood counts which might cause bias. Additionally, although we considered as many covariates as we can, there are still some confounders remain unadjusted.

## Conclusion

This study demonstrates that the inflammation index SIRI is associated with increased all-cause and CVD mortality among patients with hypertension. SIRI is considered as a potential inflammatory biomarker in the clinical practice. Further large-scale cohort studies are required to confirm our findings.

## Data availability statement

The original contributions presented in the study are included in the article/Supplementary material, further inquiries can be directed to the corresponding authors.

## Ethics statement

Ethical review and approval was not required for the study on human participants in accordance with the local legislation and institutional requirements. The patients/participants provided their written informed consent to participate in this study.

## Author contributions

SZ: data analysis, software, and writing original draft. SD: writing original draft. YQ: methodology. YW: software. BZ: writing—reviewing and editing. AL: conceptualization, funding acquisition, and writing—reviewing and editing. All authors contributed to the article and approved the submitted version.

## Supplementary material

Supplementary Figure 1 Subgroup analysis for the association between SIRI and all-cause mortality **(A)** and CVD mortality **(B)** among hypertension patients stratified by confounders.

Supplementary Table 1 The predictive ability of SIRI and traditional inflammatory biomarkers for all-cause and CVD mortality.

Supplementary Table 2 Risk of all-cause and CVD mortality among hypertension patients according to SIRI after exclusion died within 2 years of follow-up.

Supplementary Table 3 Risk of all-cause and CVD mortality among hypertension patients according to SIRI after exclusion abnormal blood data.

Supplementary Table 4 Risk of all-cause and CVD mortality among hypertension patients according to SIRI.

Supplementary Table 5 Risk of all-cause mortality among hypertension patients according to tertiles of SIRI.

Supplementary Table 6 Risk of all-cause mortality among hypertension patients according to monocyte.
