# Supplementary figures and images for: Inflammation index SIRI is associated with increased all-cause and cardiovascular mortality among patients with hypertension
Source: Front Cardiovasc Med. 2023 Jan 11;9:1066219. doi: 10.3389/fcvm.2022.1066219 (PMC9874155; doi:10.3389/fcvm.2022.1066219)

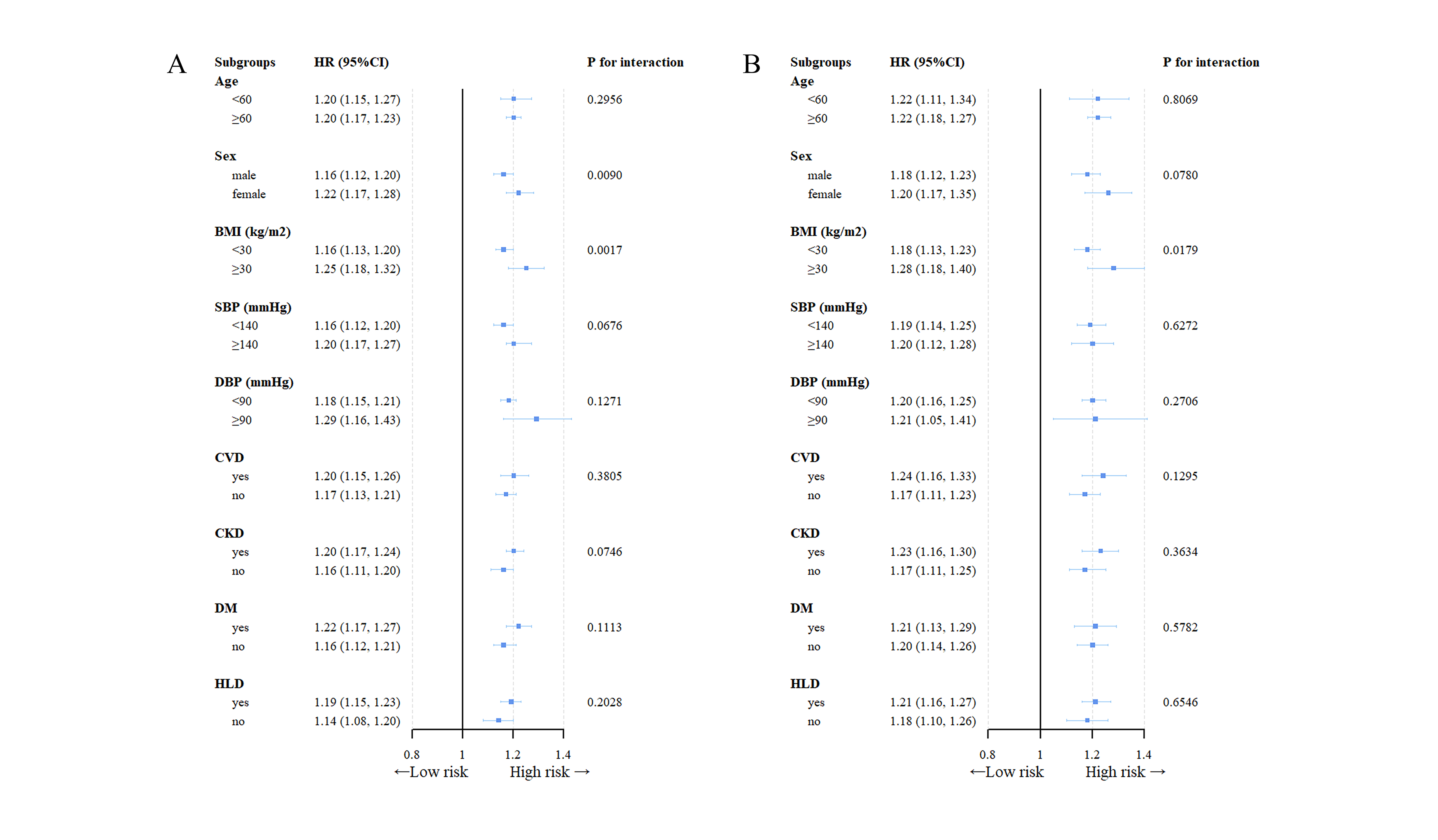

Supplement: Supplementary Figure 1 — Subgroup analysis for the association between SIRI and all-cause mortality (A) and CVD mortality (B) among hypertension patients stratified by confounders. [file Image_1.TIF]
