# Supplementary material for: Inflammation index SIRI is associated with increased all-cause and cardiovascular mortality among patients with hypertension
Source: Front Cardiovasc Med. 2023 Jan 11;9:1066219. doi: 10.3389/fcvm.2022.1066219 (PMC9874155; doi:10.3389/fcvm.2022.1066219)
Supplement: Supplementary Table 1 — The predictive ability of SIRI and traditional inflammatory biomarkers for all-cause and CVD mortality. [file Table_1.docx]

Supplementary Material

Supplementary Table 1 The Predictive Ability of SIRI and traditional inflammatory biomarkers for all-cause and CVD mortality.

|  | AUC | 95% CI | Sensitivity | Specificity | Youden index | Cut-off point |
| --- | --- | --- | --- | --- | --- | --- |
| **All-cause mortality** |  |  |  |  |  |  |
| SIRI | 0.616 | 0.608-0.625 | 0.611 | 0.562 | 0.174 | 1.231 |
| NLR | 0.613 | 0.604-0.621 | 0.692 | 0.487 | 0.179 | 2.408 |
| M | 0.555 | 0.547-0.564 | 0.521 | 0.561 | 0.082 | 0.550 |
| PLR | 0.565 | 0.556-0.574 | 0.797 | 0.310 | 0.106 | 159.045 |
| **CVD mortality** |  |  |  |  |  |  |
| SIRI | 0.607 | 0.593-0.620 | 0.583 | 0.580 | 0.163 | 1.231 |
| NLR | 0.605 | 0.591-0.618 | 0.560 | 0.598 | 0.158 | 2.239 |
| M | 0.547 | 0.534-0.560 | 0.365 | 0.707 | 0.072 | 0.650 |
| PLR | 0.556 | 0.543-0.570 | 0.433 | 0.664 | 0.096 | 139.258 |

N: neutrophil; M: monocyte, NLR: neutrophil-lymphocyte ratio; PLR: platelet-lymphocyte ratio.

Supplementary Table 2 Risk of all-cause and CVD mortality among hypertension patients according to SIRI after exclusion died within 2 years of follow-up.

|  | Model 1 | Model 2 | Model 3 |
| --- | --- | --- | --- |
|  | HR (95% CI) | HR (95% CI) | HR (95% CI) |
| All-cause mortality |  |  |  |
| Continuous | **1.29 (1.25, 1.34)** | **1.19 (1.14, 1.24)** | **1.18 (1.14, 1.21)** |
| Categories |  |  |  |
| Q1 | Reference | Reference | Reference |
| Q2 | **1.21 (1.08, 1.35)** | 1.10 (0.99, 1.22) | 1.06 (0.95, 1.17) |
| Q3 | **1.60 (1.43, 1.79)** | **1.32 (1.19, 1.46)** | **1.19 (1.08, 1.32)** |
| Q4 | **2.56 (2.28, 2.88)** | **1.83 (1.64, 2.05)** | **1.53 (1.37, 1.69)** |
| P for trend | <0.0001 | <0.0001 | <0.0001 |
| CVD mortality |  |  |  |
| Continuous | **1.31 (1.25, 1.36)** | **1.19 (1.13, 1.26)** | **1.20 (1.15, 1.25)** |
| Categories |  |  |  |
| Q1 | Reference | Reference | Reference |
| Q2 | 1.03 (0.87, 1.22) | 0.93 (0.80, 1.09) | 0.91 (0.78, 1.07) |
| Q3 | **1.47 (1.19, 1.81)** | 1.19 (0.98, 1.44) | 1.07 (0.89, 1.29) |
| Q4 | **2.75 (2.27, 3.34)** | **1.90 (1.56, 2.31)** | **1.60 (1.32, 1.95)** |
| P for trend | <0.0001 | <0.0001 | <0.0001 |

Model 1: unadjusted. Model 2: age, sex, race Model 3: further adjusted for education, income to poverty, BMI, SBP, DBP, smoking status, drinking status, CVD, CKD, DM, and HLD.

Supplementary Table 3 Risk of all-cause and CVD mortality among hypertension patients according to SIRI after exclusion abnormal blood data.

|  | Model 1 | Model 2 | Model 3 |
| --- | --- | --- | --- |
|  | HR (95% CI) | HR (95% CI) | HR (95% CI) |
| All-cause mortality |  |  |  |
| Continuous | **1.45 (1.37, 1.53)** | **1.29 (1.25, 1.34)** | **1.23 (1.19, 1.28)** |
| Categories |  |  |  |
| Q1 | Reference | Reference | Reference |
| Q2 | **1.22 (1.08, 1.37)** | 1.08 (0.96, 1.21) | 1.04 (0.93, 1.16) |
| Q3 | **1.64 (1.46, 1.85)** | **1.30 (1.17, 1.44)** | **1.22 (1.10, 1.35)** |
| Q4 | **2.73 (2.42, 3.08）** | **1.77 (1.59, 1.98)** | **1.51 (1.36, 1.67)** |
| P for trend | <0.0001 | <0.0001 | <0.0001 |
| CVD mortality |  |  |  |
| Continuous | **1.52 (1.41, 1.65)** | **1.36 (1.29, 1.44)** | **1.31 (1.19, 1.28)** |
| Categories |  |  |  |
| Q1 | Reference | Reference | Reference |
| Q2 | 1.09 (0.92, 1.30) | 0.92 (0.78, 1.09) | 0.92 (0.77, 1.10) |
| Q3 | **1.60 (1.30, 1.97)** | 1.16 (0.97, 1.40) | 1.09 (0.90, 1.31) |
| Q4 | **3.32 (2.74, 4.02)** | **1.78 (1.47, 2.14)** | **1.53 (1.26, 1.84)** |
| P for trend | <0.0001 | <0.0001 | <0.0001 |

Model 1: unadjusted. Model 2: age, sex, race Model 3: further adjusted for education, income to poverty, BMI, SBP, DBP, smoking status, drinking status, CVD, CKD, DM, and HLD.

Supplementary Table 4 Risk of all-cause and CVD mortality among hypertension patients according to SIRI

|  | Model 4 | Model 5 |
| --- | --- | --- |
|  | HR (95% CI) | HR (95% CI) |
| All-cause mortality |  |  |
| Continuous | **1.18 (1.14, 1.21)** | **1.23 (1.18, 1.28)** |
| Categories |  |  |
| Q1 | Reference | Reference |
| Q2 | 1.03 (0.93, 1.14) | 0.94 (0.76, 1.10) |
| Q3 | **1.17 (1.06, 1.28)** | **1.31 (1.10, 1.52)** |
| Q4 | **1.55 (1.41, 1.71)** | **1.51 (1.33, 1.76)** |
| P for trend | <0.0001 | <0.0001 |
| CVD mortality |  |  |
| Continuous | **1.30 (1.22, 1.39)** | **1.24 (1.19, 1.30)** |
| Categories |  |  |
| Q1 | Reference | Reference |
| Q2 | 0.93 (0.79, 1.09) | 0.94 (0.78, 1.13) |
| Q3 | 1.07 (0.90, 1.28) | **1.28 (1.06, 1.54)** |
| Q4 | **1.61 (1.34, 1.94)** | **1.53 (1.31, 1.78)** |
| P for trend | <0.0001 | <0.0001 |

Model 4: adjusted for age, sex, race, education, income to poverty, BMI, SBP, DBP, smoking status, drinking status, CVD, CKD, DM, HLD, and use of anti-hypertensive, hypoglycemic, and lipid-lowering medications. Model 5: model 3 further adjusted for CRP (mg/dl).

Supplementary Table 5 Risk of all-cause mortality among hypertension patients according to tertiles of SIRI

|  | Model 1 | Model 2 | Model 3 |
| --- | --- | --- | --- |
|  | HR (95% CI) | HR (95% CI) | HR (95% CI) |
| All-cause mortality |  |  |  |
| T1 | Reference | Reference | Reference |
| T2 | **1.30 (1.19, 1.43)** | 1.14 (1.05, 1.25) | 1.08 (0.99, 1.32) |
| T3 | **2.30 (2.09, 2.54)** | **1.71 (1.56, 1.87)** | **1.44 (1.32, 1.57)** |
| P for trend | <0.0001 | <0.0001 | <0.0001 |
| All-cause mortality |  |  |  |
| T1 | Reference | Reference | Reference |
| T2 | **1.34 (1.13, 1.58)** | **1.17 (1.00, 1.37)** | 1.11 (0.95, 1.29) |
| T3 | **2.59 (2.21, 3.04)** | **1.85 (1.58, 2.17)** | **1.53 (1.31, 1.78)** |
| P for trend | <0.0001 | <0.0001 | <0.0001 |

Model 1: unadjusted. Model 2: age, sex, race Model 3: further adjusted for education, income to poverty, BMI, SBP, DBP, smoking status, drinking status, CVD, CKD, DM, and HLD.

Supplementary Table 6 Risk of all-cause mortality among hypertension patients according to monocyte.

|  | Model 1 | Model 2 | Model 3 |
| --- | --- | --- | --- |
|  | HR (95% CI) | HR (95% CI) | HR (95% CI) |
| All-cause mortality | **1.68 (1.40, 2.03)** | **1.41 (1.19, 1.66)** | **1.28 (1.11, 1.47)** |
| CVD mortality | **1.67 (1.38, 2.03)** | **1.35 (1.14, 1.61)** | **1.19 (1.00,1.42)** |

Model 1: unadjusted. Model 2: age, sex, race Model 3: further adjusted for education, income to poverty, BMI, SBP, DBP, smoking status, drinking status, CVD, CKD, DM, and HLD.
